# Q&A: Expansion microscopy

**DOI:** 10.1186/s12915-017-0393-3

**Published:** 2017-06-19

**Authors:** Ruixuan Gao, Shoh M. Asano, Edward S. Boyden

**Affiliations:** 1 Media Lab, Massachusetts Institute of Technology (MIT), Cambridge, MA USA; 2 Internal Medicine Research Unit, Pfizer Inc., Cambridge, MA USA; 3 McGovern Institute, MIT, Cambridge, MA USA; 4 Department of Biological Engineering, MIT, Cambridge, MA USA; 5 Department of Brain and Cognitive Sciences, MIT, Cambridge, MA USA

## Abstract

Expansion microscopy (ExM) is a recently invented technology that uses swellable charged polymers, synthesized densely and with appropriate topology throughout a preserved biological specimen, to physically magnify the specimen 100-fold in volume, or more, in an isotropic fashion. ExM enables nanoscale resolution imaging of preserved samples on inexpensive, fast, conventional microscopes. How does ExM work? How good is its performance? How do you get going on using it? In this Q&A, we provide the answers to these and other questions about this new and rapidly spreading toolbox.

## What is expansion microscopy?

**Fig. 1.**
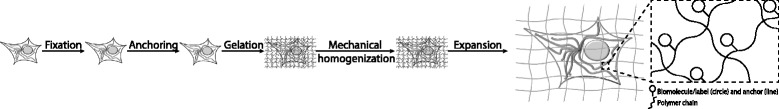
Expansion microscopy (ExM) workflow. First, a biological specimen is chemically fixed (*Fixation*). Next, the specimen is treated with compounds that bind to key biomolecules or labels (*Anchoring*), so they can be tethered to the hydrogel polymer chains synthesized in the next step. A hydrogel made of closely spaced, densely cross-linked, highly charged monomers is then polymerized evenly throughout the cells or tissue, intercalating between and around the biomolecules or labels (*Gelation*). Then the embedded specimen goes through a mechanical homogenization step involving denaturation and/or digestion of structural molecules (*Mechanical homogenization*). The specimen–hydrogel composite is now ready for physical expansion by dialysis in low-salt buffer or water (*Expansion*). Biomolecules or labels of interest remain bound to the expanded polymer network, which has pulled them apart (as schematized in the *dashed box*). Images were adapted by permission with Macmillan Publishers Ltd: Nature Biotechnology [4], copyright 2016

Expansion microscopy (ExM) is a recently-developed technique that physically expands preserved cells and tissues isotropically via a chemical process, so that 3-D nanoscale resolution imaging of specimens becomes possible on common, fast, diffraction-limited microscopes. The basic discovery of isotropic specimen expansion was first reported in 2015 [1]. In ExM (Fig. 1), biomolecules or labels are chemically equipped with anchors that enable them to be bound to a web of swellable polymer (or hydrogel) that is synthesized densely and evenly throughout the specimen (a process called polymerization or gelation). The spacing between polymers is estimated to be around a few nanometers, in the ballpark size of a biomolecule [2], so that expansion of the polymer pulls biomolecules or labels apart from each other. The polymer is densely cross-linked, so as the polymer threads swell apart from each other, the biomolecules or labels retain their spatial organization relative to one another. Polymer-embedded samples are mechanically homogenized (by high temperature denaturation, proteolysis, or other processes), then swollen by adding a solvent (water in the case of the sodium polyacrylate polymers used in ExM to date). Thus, biomolecules or labels initially spaced within the diffraction limit of a microscope can be pulled apart far enough to enable them to be easily resolved post-expansion. Step-by-step protocols for ExM and variants (described in detail in the answers to the following questions) have been posted at a dedicated website [3].

## Can you give me a quick overview of the variants of ExM and their performance?

**Fig. 2.**
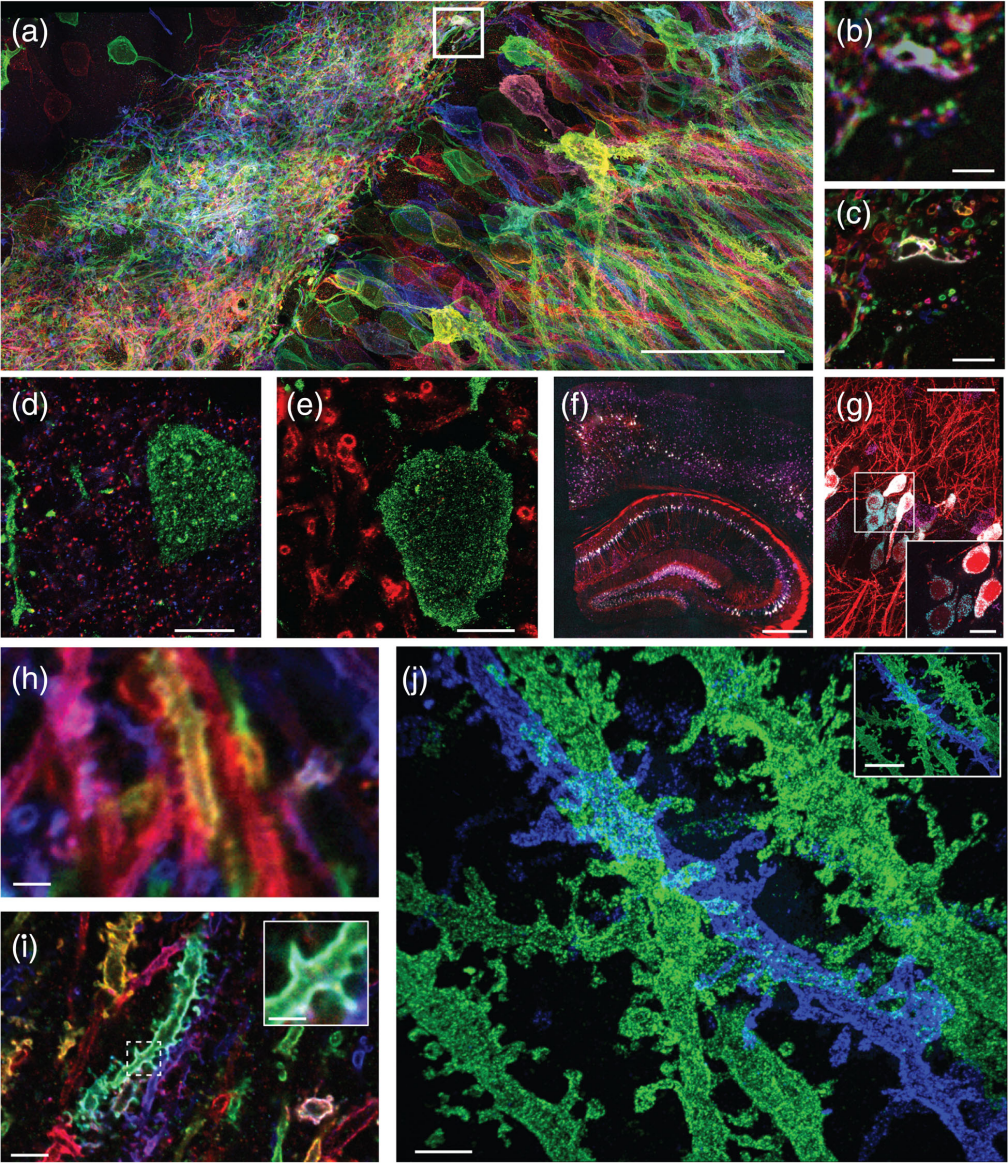
Nanoscale imaging with ExM. **a**–**c** Imaging at 60–70 nm resolution of mouse hippocampus after viral delivery of membrane-bound Brainbow3.0 followed by antibody staining, using protein retention ExM (proExM) and imaged on a confocal microscope. **a** Maximum intensity projection (MIP) of an image stack. **b** Pre-expansion image showing one optical section of the *boxed region* in **a**. **c** Post-expansion image of **b**. **d**, **e** Nanoscale imaging of mouse brain using proExM with post-expansion antibody delivery. **d** Post-expansion image of mouse cortex from Thy1-YFP mouse after high-temperature treatment, followed by immunostaining against bassoon (*blue*), homer (*red*), and YFP (*green*). **e** Post-expansion image of mouse cortex from Thy1-YFP mouse after high-temperature treatment, followed by immunostaining against myelin basic protein (*red*) and YFP (*green*). **f**, **g** Nanoscale imaging of RNA in Thy1–YFP mouse brain using expansion fluorescence in situ hybridization (ExFISH). **f** Wide-field image showing YFP protein (*red*), and hybridization chain reaction ExFISH (HCR-ExFISH) signals for YFP mRNA (*cyan*) and Gad1 mRNA (*magenta*). **g** Confocal image of mouse hippocampus from **f**. *Inset*: one plane of the *boxed region*. Colors as in **f**. **h**–**j** Imaging at ~25 nm resolution of mouse hippocampus using iterative expansion microscopy (iExM). **h** Image of unexpanded mouse hippocampus after viral delivery of membrane-bound Brainbow3.0, followed by antibody staining against EYFP (*blue*), TagBFP (*red*), and mTFP (*green*). **i** As in **h**, but expanded using proExM. *Inset*: a magnified image of spines from the *dotted box* of **i. j** MIP of an image stack of iExM-expanded mouse hippocampus with staining against EYFP (*blue*) and mCherry (*green*). *Inset*: zoomed-out view of the image of **j**. Scale bars, in biological units: **a** 50 μm (physical size post-expansion 198 μm); **b** 5 μm; **c** 5 μm (19.8 μm); **d** 5 μm (21 μm); **e** 5 μm (21 μm); **f** 500 μm (1450 μm); **g** 50 μm (145 μm), inset 10 μm (29 μm); **h** 3 μm; **i** 3 μm (14 μm), inset 1 μm (4.5 μm); **j** 1 μm (20 μm), inset 3 μm (60 μm). Images adapted by permission from Macmillan Publishers Ltd: *Nature Biotechnology* [4], copyright 2016; *Nature Methods* [5], copyright 2016; *Nature Methods* [6], copyright 2017

To tackle problems in biology, many forms of expansion microscopy have been developed, including protein retention ExM (proExM [4]; see corresponding section below and Fig. 2a–e for details) for ~100× volumetric (~4.5× linear) expansion of specimens for visualization of antibody staining and fluorescent proteins—key to mapping the architecture of signaling protein cascades within and between cells; expansion fluorescent in situ hybridization (ExFISH [5]; see section below and Fig. 2f, g) for expansion of specimens for visualization of RNA—key to understanding the organization of structural RNAs and the location of RNAs in nanoscale compartments of cells such as synapses; and iterative ExM (iExM [6]; see section below and Fig. 2h–j), in which a sample is expanded twice (that is, ~10,000× volumetric expansion) for extremely fine resolution. In 2016, other groups began to publish related variants of expansion microscopy [7–9], suggesting that the general expansion microscopy concept is robust and easy to implement and deploy. The original ExM and proExM variants generate roughly ~4.5× linear expansion, the ExFISH variant generates ~3× linear expansion (due to the buffers required for in situ hybridization), and the iExM variant expands by a linear factor of ~4.5 × ~4.5 or 20×. For a ~300 nm diffraction limit objective lens, the effective resolution for a ~4.5× expansion factor protocol would be ~300 nm/4.5 = 60–70 nm; for a ~3× expansion factor protocol, the effective resolution would be ~300/3 = 100 nm; for a ~20× expansion you would, in principle, have ~300/20 = 15 nm resolution, but in practice, for the published version of iExM, the size of the antibodies determines the limit on resolution (since they are delivered pre-expansion), and thus we obtained a resolution of 25 nm.

## Does ExM move cells apart from each other, or rip the cells apart?

This isn’t quite the right way to think about ExM. In ExM we are forming polymer meshes that have spacings in the few nanometer range. So the size scale of the polymer chains, and the spacing between them, is far smaller than the size scale of a cell. One way to think about the process is that the polymer chains, which wind their way around and between biomolecules, will pull the biomolecules apart from each other. As a result of this process, the biomolecules that make up a cell will be separated from each other. The lipid membrane will be fragmented as well. The molecules between cells will also be separated from one another. So every molecule will be moved apart from its neighbors. The process is analogous to drawing a picture on a balloon and then blowing up the balloon: the ink particles will move apart from each other, but their relative organization is the same. Of course, this is the ideal case we are describing here. If you do not mechanically homogenize the sample adequately, and it resists expansion, one can get fracturing of cells and tissues [4].

## Are there any other methods for expanding tissues in addition to ExM?

Interestingly, many protocols for tissue preparation have, as a side effect, the phenomenon of expanding tissues, although it is unknown whether these effects are isotropic, and these protocols tend to treat the effect as undesired and try to minimize it. This includes the Scale protocol for brain tissue clearing [10], the CLARITY protocol [11], and the CUBIC protocol [12], which each cause a degree of tissue expansion, at least during some steps of the protocol. In ExM, we deliberately expand biological specimens, to a large extent, and in an isotropic fashion, with accuracy down to the nanoscale [1]. ExM achieves this by embedding the specimens in hydrogels, which have a multi-decade history as a microscopy tissue preparation medium [13, 14]. In ExM, the hydrogels are designed to be extremely dense (so that nanoscale information can be captured), polyelectrolyte in nature (i.e., composed of charged building blocks) for high swelling force, and with a cross-linked topology (so that expansion will preserve the relative organization of biomolecules, when specimens are mechanically homogenized and then swollen).

## What technical problems does ExM solve?

ExM enables nanoscale resolution imaging of extended 3-D preserved specimens, like cells and tissues. Furthermore, it only requires diffraction-limited microscope hardware already common in biology labs. Earlier super-resolution methods require hardware that is complex and/or expensive. In addition, they are physically limited in imaging speed, number of colors, and/or in the volume accessible, as compared to diffraction-limited microscopy, in part because of the intrinsic physical principles governing how methods like STORM, PALM, and STED work [15–18]. ExM-processed samples, in contrast, can be imaged on fast diffraction-limited microscopes (which are getting faster, driven by ongoing engineering). Since ExM expands samples in water, final expanded specimens are ~99% water, and thus are essentially completely transparent and optical aberration free. This facilitates large-volume imaging of expanded samples with, for example, light-sheet microscopy, which can proceed orders of magnitude faster in volumetric acquisition speed than earlier microscopy technologies [5]. The net result is imaging speeds, numbers of colors, and volumes that can be many times (and sometimes orders of magnitude) greater than with classic super-resolution methods, while still obtaining images of similar or better resolution.

Another benefit of ExM is the decrowding of biomolecules or labels by expansion of them away from each other, which makes room around the molecules for chemical reactions to take place. This extra room can be used to perform signal amplification (for example, via hybridization chain reaction (HCR) [19], and potentially via rolling circle amplification (RCA) [20]), so that rather than attempting difficult single molecule imaging in 3-D volumes of intact tissue, one can attach many fluorophores to a single-molecule biomolecular target, making the identification and localization of single biomolecules feasible in large volumes (as demonstrated for HCR and ExFISH applied to the detection of single RNAs in synapses in intact brain tissue in [5], and shown in Fig. 2f, g). Decrowding molecules may also improve antibody performance, facilitating antibody diffusion into samples and access to epitopes (Fig. 2d, e) [4, 8]. A related and subtle point relates to the growing excitement about temporal multiplexing imaging strategies, where a single biomolecule must be tracked over many cycles of affinity tag binding or nucleic acid sequencing in order to identify the biomolecule accurately [20–22]. If two molecules are too close, it may be difficult to preserve their identity over many consecutive cycles of imaging; decrowding the molecules may help make this tracking more accurate [5].

## What equipment do I need for ExM?

You likely already have access to all the equipment you need for ExM. Beyond a conventional diffraction-limited microscope, you need freezers, incubators, and a few other common items. For proExM and ExFISH, you can use commercially available reagents to perform the entire process. For the original 2015 ExM protocol [1] and the iExM protocol [6], you need to attach DNA oligonucleotides to antibodies, since the DNA oligonucleotide is the actual reagent that is anchored to the swellable polymer. The DNA oligonucleotide–antibody conjugation protocol is posted at the website [3], and we will post links on this website to commercial sources for these antibodies as they become available.

## What kinds of specimens can be expanded with ExM?

A wide range of samples have been successfully expanded and imaged using ExM, including *Escherichia coli* bacteria [23], cultured mammalian cells [4, 5, 24, 25], mouse cortex, hippocampus, striatum, and other brain regions [1, 4–6, 26], lung [4], pancreas [4], spleen [4], and non-human primate brain [4], with many other species (including *Drosophila*, zebrafish, and human) and tissue types (including skin, kidney, colon, liver, and others, both normal and from disease states such as cancer) in the pipeline (as reflected by public talks, poster presentations, and bioRxiv preprints from various groups). At MIT, we regularly host people to come watch us do expansion microscopy, and perhaps hundreds of groups are now performing it. To our knowledge, expansion of wood or bone has not yet been publicly reported. But one might imagine that as long as you can form polyelectrolyte polymer chains evenly between and around biomolecules in a specimen, in a mesh-like topology, and as long as you can mechanically homogenize a specimen so that its structure does not resist expansion, the specimen might very well be expandable.

## Does ExM work on protein-dense regions like the synaptic cleft?

This is an interesting question. If we are asking the question regarding very small length scales—for example, about whether five molecules in a macromolecular complex are being moved apart from each other evenly—the answer as of today is that we have not yet validated ExM down to the few-nanometer level. However, we have validated its resolution down to around 25 nm, and at that level of resolution, indeed synapses seem to be expanded in a fashion that lets you resolve scaffolding proteins, neurotransmitter receptors, and other densely packed synaptic components [6]. But to expand on this further, there are really two questions here: first, can you label proteins that are densely packed? The answer is that you might do a better job if you expand the proteins away from each other first [4]: labels like antibodies have nonzero size, and in many cases are larger than the proteins they are binding to, meaning that crowding between labels may result in poor staining. Second, does the polymerization and expansion process evenly anchor and pull on proteins that are densely packed? We recently showed that iExM is likely spatial information-preserving down to the ~5–10 nm range [6], which is reassuring but does not guarantee that individual proteins can be pulled apart—getting down to the resolution of structural biology will require further work.

## Can you section ExM samples?

Most likely. We have not been focusing on this in our group, since we’ve been focusing more on the fundamental chemistry of ExM so far, but our guess is that sectioning expanded samples (especially if they have been re-embedded in acrylamide for post-expansion reinforcement [5]) could be performed using vibratomes or other sectioning devices. If you give this a try, or want to collaborate on an experiment to explore this hypothesis, we are happy to discuss what resources or insights we might have to offer.

## When should I use proExM?

Protein retention ExM (proExM) is a simple, yet powerful, variant of ExM, in which proteins are anchored to the swellable polymer generated during the ExM process (Fig. 2a–e) [4], via a commercially available small molecule (which we call AcX for short) that binds to amines on proteins and simultaneously to the polymer matrix. Fluorescent antibodies can be administered before (Fig. 2a–c) or after (Fig. 2d, e) expansion. In post-expansion staining proExM, all proteins in biological samples are covalently anchored to the polymer via AcX, and then gentle mechanical homogenization (for example, high temperature denaturing in detergent solution) and expansion occur, followed by staining with fluorescently labeled antibodies. The decrowding of epitopes can help provide physical space between proteins to facilitate staining. However, some epitopes may be lost during the denaturation step. A protocol similar in some regards, the magnified analysis of the proteome (MAP) protocol [8] works with ~80% of antibodies attempted. In another variant of proExM, pre-expansion staining proExM, biological samples express fluorescent proteins and/or are stained with fluorescent antibodies before the gelation process begins. The fluorescent proteins and/or antibodies, along with other proteins, are covalently anchored to the polymer, and then proteases are administered to a degree that chops up enough proteins so that mechanical homogenization occurs, but not to the point where relatively protease-resistant fluorescent proteins and/or antibodies lose their function. This is a popular methodology because it fits directly into existing workflows for antibody staining: you can take an existing antibody-stained sample and enter it into this expansion pipeline. Another group independently developed a similar protocol [7].

## What fluorescent proteins and dyes are compatible with proExM?

In post-expansion staining proExM, antibodies bearing essentially any dye should work, since the antibodies do not go through the gelation process but are simply administered after the expansion. In pre-expansion staining proExM, most fluorescent proteins and dyes are compatible (that is, there is >50% fluorescence retention), although cyanine-family dyes (such as Alexa 647 or Cy5) are degraded during the polymerization step. Therefore, for pre-expansion staining proExM, we recommend using, for example, Atto647N if a far-red dye is desired. Regarding fluorescent proteins, GFP-like fluorescent proteins are protease resistant and can survive the proExM process well (that is, with >50% fluorescence retention), but non-GFP-like fluorescent proteins (such as infrared proteins based on bacteriophytochrome) are easily destroyed by the proteinase step. For more detail, see the plots in the first figure of [4].

## When should I use ExFISH?

Expansion fluorescence in situ hybridization (ExFISH) is a version of ExM in which RNAs (and DNA, although to date we have primarily focused on RNA) in a biological sample are retained during the ExM process (Fig. 2f, g) [5]. In ExFISH, RNAs are covalently anchored to the hydrogel with a small molecule that we call LabelX (and which is made by mixing two commercially available reagents) that binds to guanine and also to the hydrogel. By applying the protein-anchoring reagent (AcX) simultaneously, you can anchor both RNAs and proteins to the hydrogel for dual protein/RNA visualization. For imaging, RNAs can be labeled with FISH probes after expansion. As noted earlier, ExFISH lends itself easily to post-expansion fluorescence amplification by applying HCR, so that rather than imaging single or small numbers of fluorophores in intact tissue, each biomolecular target is coupled to dozens or perhaps hundreds of fluorophores, for easier detection, even in nanoscale compartments like dendritic spines in intact mouse brain circuitry. In the future, ExFISH may help with temporally multiplexed FISH probing, which has recently been adapted for use with intact tissues [27, 28].

## What is iExM and when should I use it?

We recently introduced the concept of iterative ExM (iExM) (Fig. 2h–j) [6], where a biological specimen is first expanded by ExM, then a second swellable gel is formed in the space opened up by the first expansion, and then the sample is expanded a second time. This double-expansion process results in a linear expansion factor of about ~4.5 × 4.5 = 20× and an effective resolution of ~25 nm after two rounds of expansion (larger than the expected ~300 nm/20 = 15 nm due to the size of the antibodies and DNA linkers). iExM is sufficient for resolving proteins within synapses in 3D, as well as very fine parts of neurons (such as dendritic spine necks (Fig. 2j)).

## How isotropic is the expansion in ExM, and what is the limit on resolution?

We have quantified, for each ExM variant, the isotropy of the expansion process. One way to do that is to take pre-expansion images on a diffraction-limited or classic super-resolution microscope (for example, a SIM or STORM microscope), and then take post-expansion images on a conventional diffraction-limited microscope. By performing a non-rigid registration of the pre- and post-expansion images with respect to each other (after they’ve been aligned as much as possible through the rigid transformations of rotation, scaling, and translation), the amount of distortion between the two images can be calculated. Across a wide variety of cell types and tissues, the published ExM protocols from us (and now others) have shown distortions in length of 1–4% across length scales of tens to hundreds of microns [1, 4–8].

The resolution of ExM can be estimated by taking post-expansion images of objects of known size, such as microtubules. By deconvolving the size of a feature (in this case, microtubule diameter [1] or sidewall width [6]) as it appears in ExM, by the “ground truth” feature size obtained previously by electron microscopy (with ExM labels such as antibodies added in simulation), we obtained effective resolutions of ~60–70 nm for ~4.5× forms of expansion [1] and ~25 nm for ~20× forms of expansion [6]. Compare these numbers to those obtained in the earlier section “Can you give me a quick overview of the variants of ExM and their performance?”. Recently, we estimated that the amount of error introduced by the two rounds of gelation and expansion in iExM (ignoring the contributions of antibodies, optical diffraction, and so forth) was on average around 5–10 nm [6], suggesting that the properties of the polymer are such that potentially extremely good resolutions are possible with some fine tuning of the process.

## Can I shrink expanded samples back down?

Yes, the expansion can be reversed by adding salt to collapse the expanded polymer back into the shrunken state. We often shrink ExM samples from the fully expanded state so we can store them more stably, in a buffered environment (for example, we often use phosphate-buffered saline (PBS)) that is more controlled and better for biomolecular stability than a pure-water, unbuffered, environment. A buffered environment prevents fluorescent proteins from degrading or denaturing and also helps keep FISH probes hybridized to nucleic acids in ExFISH samples [5]. Shrinking a sample can also help reduce the imaging volume: if you only need a 2× or 3× expansion, and not a full ~4.5× expansion, for your scientific question, using salt to reduce the imaging volume can enable the process of imaging to go faster.

## How do I handle ExM samples?

Expanded samples are hydrogels that are mostly water, and thus require gentle handling. They are robust to some extent, but are definitely more fragile in the expanded state than in the shrunken state in PBS. We recommend handling ExM samples as much as possible in the shrunken state. Paintbrushes can easily be used to handle gels in PBS: insert the paintbrush below the gel, lift it up, and flip it (if it has been inverted) or transfer it to the target container (see our video tutorial at [29]). Alternatively, flexible plastic spatulas can be used to scoop up such gels. When it is necessary to handle ExM samples in their expanded state (such as when mounting the expanded samples, with the techniques described later), you may not want to use a paintbrush to pick up the gel. Alternatively, you might consider using a coverslip to carry the expanded gel. Place a thin (No. 1) coverslip next to the gel, then slide the gel on top of the coverslip with a paintbrush. Remove excess liquid from around the gel so it won’t slide off the coverslip. Finally, use a pair of forceps to carry the coverslip to the target container, and then slide the gel off the coverslip into the target container by gently pushing with the paintbrush.

## Where is my ExM sample? It’s so clear I can’t find it

**Fig. 3.**
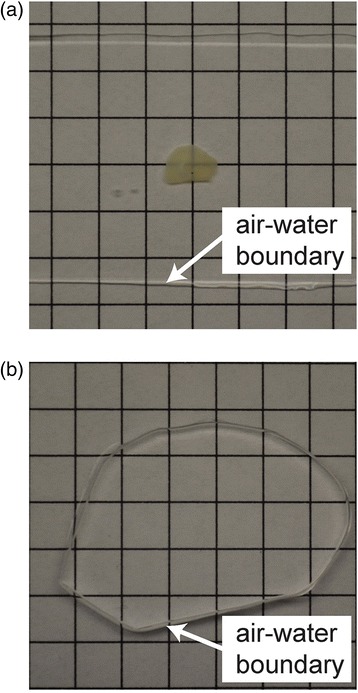
After expansion, the sample is 99% water and clear. Expansion significantly reduces scattering of the sample since the sample is mostly water. A 200-μm fixed brain slice is opaque primarily due to scattering (**a**). However, the post-ExM sample is transparent (**b**), facilitating light-sheet imaging and large-volume imaging. From [1]. Reprinted with permission from AAAS

After expansion, the refractive index of ExM samples becomes essentially the same as that of water (Fig. 3). It may be hard to find your sample with bare eyes, or with bright-field imaging. If you remove excess water, you will be able to see the contour of the gel. If you trim your gel to an easy-to-recognize shape before expanding it, you will easily be able to tell the orientation of your sample, and also whether it is flipped over or not (see our video tutorial at [29]). Be sure to keep your gel hydrated to prevent collapse. We recommend initially imaging with a low magnification fluorescence microscope, such as a dissection scope or a wide-field epifluorescence microscope with low magnification and long working distance (4× or 10×) objectives, to get your bearings when beginning to observe an expanded sample. If you still cannot find your sample in the gel, here are some things you might consider:

First, is your sample facing the objective lens, or is it on the far side of the piece of gel you are imaging? If your sample is located on the side of gel farther from the objective, it may not be within the working distance of the objective. In this case, you might need to flip the gel over so that your sample is close to the objective lens. For inverted microscopes, you might try to remove the liquid around the gel to reduce the distance between the gel and the coverslip, both to get the sample within the working distance of the objective and to make sure the sample doesn’t drift during imaging. However, even with most of the liquid removed, the gel still might drift over time—so if you want to image a sample over a long timescale (>5 min) or would like to obtain a z-stack of images, we recommend placing your sample on a poly-lysine modified surface (or mounted in another stable fashion; see next section for details). Another benefit of poly-lysine (or other style of) mounting is that it will further decrease the distance between the gel and the coverslip so that the sample is more easily placed within the objective working distance. For upright microscopes, you may need to adjust the stage or objective height over a substantial length to find the specimen.

## Do I need to mount my expanded ExM samples in my microscope?

Mounting an expanded ExM sample on a stable surface can help prevent the sample from moving during imaging, important for obtaining high quality images. The mounting method best for you will depend on the geometries of the microscope and the sample holder (coverslip, Petri dish, or well of a multiwell plate, for example), as well as your imaging requirements such as the objective lens magnification and imaging duration. For example, when you are using an inverted or upright microscope with low magnification dry objectives (between 4× and 10×) to check how the sample looks, for a short period of time (<5 min), there is probably no need to attach the gel to the sample holder: you can temporarily remove excess water from around the gel, which is often sufficient to prevent the gel from sliding during a short imaging period. But if you are using an upright microscope with a water immersion objective, water is necessary for imaging, and thus to prevent the gel from floating away you might want to mount the gel to the sample holder more enduringly. For long-term imaging (>5 min) or imaging that would be disrupted by even a little sample drift (for example, obtaining a z-stack) on an inverted, upright or light-sheet microscope, you might also want to mount the sample on a mechanically stable surface such as a coverslip (or other comparable sample holders that suit your microscope geometries).

For inverted microscopes, you can use poly-lysine to mount the gel to a stable surface (see next question for details of mounting methods). For upright microscopes, you can use agarose, poly-lysine, or superglue. For light-sheet microscopes, you can use superglue or poly-lysine. The advantage of the agarose method is that the sample mounting is reversible, so that you can retrieve your sample after imaging: you can shrink the expanded gel in high salt buffer, then carefully separate it from the agarose for storage. The advantage of the poly-lysine method is that it provides a transparent interface between the gel and the coverslip. Therefore, it is suitable for microscope configurations that require imaging through the coverslip (for example, using an inverted microscope to image an expanded sample on top of a coverslip). In such a situation, agarose situated in between the objective lens and the gel might introduce optical scattering and/or aberration. A similar issue holds for superglue, which is not transparent after hardening. The advantage of the superglue method is its strong adhesion: it can stabilize samples for imaging for long periods of time, even days.

## How do I mount expanded ExM samples in my microscope?

For inverted microscopes, we recommend using the poly-lysine method for mounting, as the inverted configuration requires imaging the expanded sample through the coverslip (or other sample holder, such as a glass-bottom multi-well plate or Petri dish). For commercial light-sheet microscopes (such as the Zeiss Z.1), in which the samples are mounted by hanging from above, we recommend using either the superglue or the poly-lysine method. You can first mount the samples using superglue or poly-lysine to a coverslip (or other thin and rigid backing of choice). Then you can attach the coverslip to the sample rod hanging from above. In our setup, we 3-D print an adaptor that can be mechanically attached to the sample rod and then we superglue the coverslip bearing the gel to the adaptor. For upright microscopes, we have successfully used all three methods including the agarose, poly-lysine, and superglue methods.

In detail, for the agarose method: dissolve low melting point agarose in water and cool to slightly above the hardening temperature. Remove excess water from around the gel. Using a pipette, drop agarose on the edges of the gel (which is on top of a sample holder such as a coverslip) and let harden. If needed, add additional agarose to cover the entire gel. Once the agarose hardens, add more water to prevent dehydration.

For the poly-lysine method: clean the sample holder surface (for example if using a coverslip, wash it with water and ethanol) and apply poly-lysine. You can follow protocols of your choice, but we find that a 20-min application of 0.1% (w/v) poly-lysine in water at room temperature, followed by three brief rinses with water, and a final drying in air, works well. Remove excess water from the gel to ensure attachment in the next step (one approach is to wick away all the excess water from the gel surface using a paper wipe). Then, transfer the gel onto the poly-lysine-modified surface. To avoid dehydration, the drying and the transfer should be done within a few minutes. After the gel is placed on the poly-lysine modified surface, gently press the gel with a soft paintbrush to facilitate adhesion. Finally, add water to avoid dehydration.

For the superglue method: apply superglue on the surface of the sample holder and use a paper wipe to soak up excess superglue, leaving a uniform thin layer. As in the poly-lysine method, water needs be wicked with a paper wipe away from the gel surface to enable successful attachment. Transfer the gel onto the area of the sample holder covered with superglue. When the superglue hardens, it will become opaque (typically in 20–30 s). Add water to avoid dehydration.

## How should I store ExM samples?

Storing samples in the expanded state for long periods of time is not advised, as noted earlier. Instead, ExM samples are typically stored in the shrunken state, in buffer such as PBS, and at 4 °C in the dark to preserve fluorescence. We have stored proExM samples, which were digested but not expanded, in this state for weeks before expansion for imaging. As noted earlier, repeatedly expanding then shrinking specimens is possible, which may help support imaging over multiple sessions. For some biomolecules, such as fluorescent proteins, storage in pure water may denature them over periods of a few hours to days. Indeed, some have seen that fluorescent proteins may become dimmer over a period of even a few hours of imaging in pure water. We recommend imaging samples containing fluorescent proteins as rapidly as possible, immediately after the initial expansion is complete. If you are looking at fluorescent proteins in large samples, we recommend staining the fluorescent proteins with fluorescent antibodies since these are robust over extended imaging periods, and can easily survive many rounds of expansion and shrinking.

## Does ExM work on live specimens?

No. If you separate proteins from each other, using the protein-preserving form of the proExM protocol, in principle some of them might still function. But lipid membranes will have been fragmented by expansion, so organelles will no longer be well-isolated compartments. Furthermore, all protein concentrations will be diluted 100×, and diffusion of proteins will no longer occur since the proteins will be attached to the polymer backbone. So in general, most living processes would be halted by expansion. But potentially, specific signaling functions or enzymatic functions, if carefully thought out and examined with appropriate controls, and potentially with modification of the ExM protocol, might be possible. For example, it might be possible to use the ExM polymer to apply force to different proteins within a multiprotein complex, and measure the force that it takes to separate them, in different physiological states. If you give this a try, or want to collaborate on uses of expansion polymers to examine protein–protein interactions as a function of physiological state, we are happy to discuss what resources or insights we might have to offer.
